# Zr(HSO_4_)_4_: Green, efficient and reusable catalyst for one-pot synthesis of 1,8-dioxooctahydroxanthene under solvent-free conditions

**DOI:** 10.1016/j.mex.2022.101832

**Published:** 2022-08-27

**Authors:** Khalil Pourshamsian

**Affiliations:** Department of Chemistry, Tonekabon Branch, Islamic Azad University, Tonekabon, Iran

**Keywords:** Green, Highly efficient, One-pot synthesis, 1,8-dioxooctahydroxanthenes, Reusable heterogeneous catalyst, Solvent-free conditions

## Abstract

Many methods have been used to synthesize xanthene derivatives using different catalysts. However, some of these methodologies have not been entirely satisfactory. Most of the mentioned methods have disadvantages such as low yields, prolonged reaction times, harsh reaction conditions and the requirement of expensive catalysis and use of toxic organic solvent. In this research, a green and highly efficient procedure for the one-pot synthesis of 1,8-dioxo-octahydro-xanthenes has been developed. Zr(HSO_4_)_4_ catalyst was used as an efficient and recoverable catalyst for synthesis of 1,8-dioxo-octahydro-xanthene derivatives *via* cyclocondensation of dimedone and aromatic aldehydes in solvent-free conditions. There are no examples of the use of Zr(HSO_4_)_4_ for the synthesis of 1,8-dioxo-octahydro-xanthene derivatives. The present method offers several advantages such as green, highly efficient, recoverable, reusable, simple work-up and simple purification of products. The structure of the synthesized products was confirmed by Fourier Transform Infrared (Ft-IR) and Proton nuclear magnetic resonance (^1^HNMR) analyzes. The antibacterial activity of the synthesized compounds was determined by agar disk diffusion method against gram-positive (S. aureus bacteria) and gram-negative (E. coli bacteria) microorganisms. Among the synthesized compounds (3a-3j), 3h compound showed the highest antibacterial effect by forming an inhibitory diameter zone of 15 mm around the disc containing 2000 mg of 3h-compound against gram-positive (S. aureus bacteria).

1. Use of Zr(HSO_4_)_4_ as a green and highly efficient and reusable heterogeneous catalyst.

2. Under solvent-free condition.

3. Simple work-up and Simple purification of products.

## Graphical abstract

**Figure:**
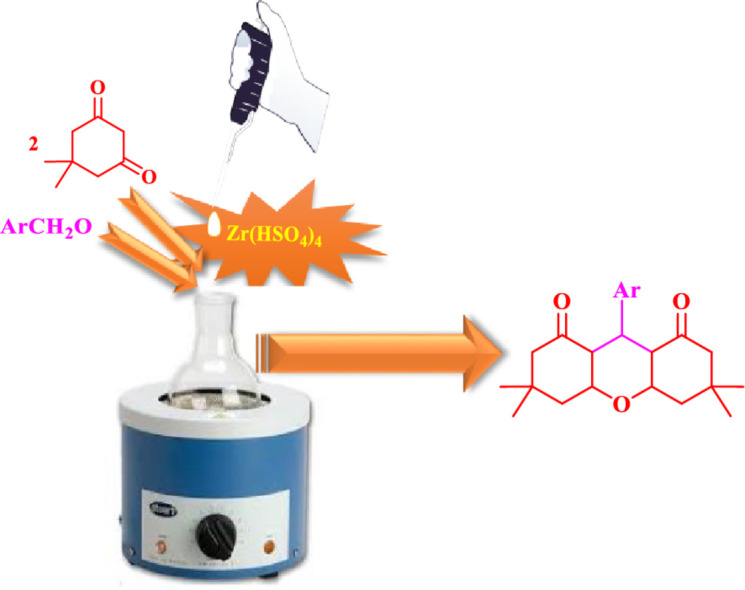

**Specifications table**

**Table:** 

Subject Area:	*Chemistry*
More specific subject area:	*Medicinal Chemistry*
Method name:	*Aldol condensation reaction*
Name and reference of original method:	*Not applicable*
Resource availability:	*Not applicable*

**Method details**

## Introduction

In recent years, synthetic chemists have shown tremendous interested in developing highly efficient transformation for the synthesis of xanthene derivatives due to their potential applications in the biological activities, pharmaceutical and industrial applications. These compounds have antibacterial properties due to the presence of reactive oxygen in their structure. For example, they have many biological and therapeutic properties such as anti-inflammatory [1], antiviral [2], antibacterial [3], as well as in photodynamic therapy [4] and as antagonists of the paralyzing action of zoxazolamine [5]. Xanthenes are also available from natural sources [6]. Many procedures are disclosed to synthesize xanthenes and benzoxanthenes such as montmorillonite K10 [7], nano-ZnAl_2_O_4_ [8], sodium hydrogen sulfate [9], cyclodehydrations [10], [C_4_dabco][BF_4_] ionic liquid [11], Smcl_3_ [12], acid activated clay [13], sulfonic acid functionalized silica [14], template-containing Zn/MCM-41 [15], Sulfamic acid [16], Nickel-Cobalt Ferrite [17], Many different methods have been reported for the synthesis of xanthenes; one of them is the condensation of aldehydes with cyclohexane-1,3-dione or 5,5-dimethylcyclohexane-1,3-dione to give 1,8-dioxooctahydroxanthene derivatives. This reaction has been conducted in the presence of strong protonic acids [18], Lewis acids such as InCl_3_.4H_2_O [19], FeCl_3_.6H_2_O [20] and heterogeneous catalysts like Dowex-50W [21], NaHSO_4_.SiO_2_ [22], Ambertyst-15 [23], HBF_4_/SiO_2_ [24] and Sulfonic acid-functionalized LUS-1 [25]. Other catalysts such as alum-promoted [26], potash alum [27], boric acid [28], ZrOCl_2_.8H_2_O [29], nano-Fe_3_O_4_ [30], B(HSO_4_)_3_ [31], TiO_2_ [32], nano-TiO_2_ [33], BiVO_4_-NPs [34] and InCl_3_ [35] and etc. [[36], [37], [38]].

However, some of these methodologies have not been entirely satisfactory. Most of the mentioned methods have disadvantages such as low yields, prolonged reaction times, harsh reaction conditions and the requirement of expensive catalysis and use of toxic organic solvent. Therefore, the use of Zr (HSO4) 4 as a heterogeneous catalyst has been considered in this research to avoid these limitations and to create an alternative pathway for the synthesis of xanthene derivatives. Zr(HSO_4_)_4_ is a low-cost solid Brønsted acid. There are no examples of the use of Zr(HSO_4_)_4_ for the synthesis of 1,8-dioxooctahydroxanthene derivatives. In this research, Zr(HSO_4_)_4_ catalyst was used as an efficient, recoverable and reusable catalyst for synthesis of 1,8-dioxooctahydroxanthene derivatives *via* traditional route from cyclocondensation of dimedone and aromatic aldehydes in solvent-free conditions.

## Experimental

All chemicals used were of synthetic grade and were used as received without any further purification. Melting points were determined in open glass capillaries on an Electrothermal 9100s apparatus and are uncorrected.

The IR spectra were recorded with FT-IR Shimadzu IR-470 instrument using potassium bromide pellets. The ^1^H-NMR spectra were determined on a Bruker Avance DRX-400 MHz instrument using TMS as the internal standard and CDCl_3_ as solvent. Chemical shifts are expressed as δ(ppm) and the coupling constant as *J*(H_2_). The progress of reaction was monitored by Thin-layer chromatography (TLC) using 0.2 mm Merck silicagel GF254 pre-coated plates and visualized by UV-light (254 nm).

Scheme 1

***General procedure for the preparation of 1,8-dioxooctahydroxanthene* (3a-j)**: To a mixture of dimedone 1 (2 mmol) and aldehyde 2 (1 mmol) was added Zr(HSO_4_)_4_ which prepared as reported [1] (20 mol%) and the mixture was allowed to stir at 110 ^°^C for the total recorded time which indicated in Table 1. The progress of reactions was monitored by TLC (eluent: EtOAc/ Diethylether, 1:4). After completion of the reaction, the reaction mixture was cooled to room temperature. Then to mixture was added cold water and the product was extracted with ethyl acetate (3 × 5 mL). The organic layer was dried (MgSO_4_) and evaporated in vacuum. The crude product recrystallized by EtOH 96% and purified. All the desired products were characterized by comparison of their physical data with those reported compounds. For the total confirmed of synthetic compounds, the spectral data given below.

**Table 1:** Screening of the catalyst and solvent for the reaction of benzaldehydes and dimedone catalyzed by Zr(HSO_4_)_4_.^a^


Entry	Catalyst (mol%)	Solvent/condition	Time (min)	Yield^b^ (%)
1	20	water/reflux	120	trace
2	20	THF/reflux	120	trace
3	20	CHCl_3_/reflux	120	20
4	20	CH_3_CN/reflux	120	15
5	20	CH_3_CH_2_OH/reflux	100	55
6	10	solvent-free	100	68
7	20	solvent-free	40	86
8	30	solvent-free	40	75
9	0	solvent-free	40	trace

a Reaction condition: dimedone (2 mmol), aldehyde (1 mmol) and Zr(HSO_4_)_4_ as catalyst.

b Isolated yield.

***3,3,6,6-tetramethyl-9-phenyl-1,8-dioxooctahydroxanthene* (3a)**: White powder, Yield (86%), mp 201-202 °C. FT-IR (ῡ, Cm^−1^) (KBr disc): 3040 (CH_arom_, Str.); 2990 (CH_aliph_, Str.); 1605 (C=O Str.); 1540 (C=C Str.); 1360 (C-O Str.). ^1^H NMR (400 MHz, CDCl_3_) δ(ppm): 1.12 (6H, s, 2CH_3_); 1.26 (6H, s, 2CH_3_); 2.30-2.50 (8H, m, 4CH_2_); 5.58 (1H, s, CH); 7.11-7.13 (2H, d, CHO, ^3^*J* = 6.8 Hz); 7.17-7.21 (1H, t, H_p_, ^3^*J* = 7.8 Hz); 7.27-7.31 (2H, dd, H_m_, ^3^*J* = 7.8 Hz, ^3^*J* = 6.8 Hz).

***3,3,6,6-tetramethyl-9-(4-methylphenyl)-1,8-dioxooctahydroxanthene* (3b)**: White powder, Yield (81%), mp 173-175 °C. FT-IR (ῡ, Cm^−1^) (KBr disc): 3080 (CH_arom_, Str.); 2950 (CH_aliph_, Str.); 1670 (C=O Str.); 1540 (C=C Str.); 1380 (C-O Str.). ^1^H NMR (400 MHz, CDCl_3_) δ(ppm): 1.01 (6H, s, CH_3_); 1.11 (6H, s, CH_3_); 2.15 (4H, dd, CH_2_); 2.26 (3H, s, CH_3_); 2.47 (4H, s, CH_2_); 4.8 (1H, s); 7.02 (2H, d, CH_arom_, ^3^*J* = 8.2 Hz); 7.19 (2H, d, CH_arom_, ^3^*J* = 8.2 Hz).

***3,3,6,6-tetramethyl-9-(4-methoxyphenyl)-1,8-dioxooctahydroxanthene* (3c)**: Gray powder, Yield (82%), mp 238-239 °C. FT-IR (ῡ, Cm^−1^) (KBr disc): 3080 (CH_arom_, Str.); 3000 (CH_aliph_, Str.); 1685 (C=O Str.); 1530 (C=C Str.); 1380 (C-O Str.). ^1^H NMR (400 MHz, CDCl_3_) δ(ppm): 1.01 (6H, s, CH_3_); 1.11 (6H, s, CH_3_); 2.22 (4H, dd, CH_2_); 2.47 (4H, s, CH_2_); 2.47 (1H, s, CH); 3.74 (3H, s, OCH_3_); 6.7 (2H, d, CH_arom_, ^3^*J* = 8.4 Hz); 7.21 (2H, d, CH_arom_, ^3^*J* = 8.4 Hz).

***3,3,6,6-tetramethyl-9-(3-methoxyphenyl)-1,8-dioxooctahydroxanthene* (3d)**: White powder, Yield (92%), mp 159-160 °C. FT-IR (ῡ, Cm^−1^) (KBr disc): 3100 (CH_arom_, Str.); 3000 (CH_aliph_, Str.); 1580 (C=O Str.); 1520 (C=C Str.); 1370 (C-O Str.). ^1^H NMR (400 MHz, CDCl_3_) δ(ppm): 1.12 (6H, s, CH_3_); 1.25 (6H, s, CH_3_); 2.30-2.48 (8H, m, CH_2_); 3.75 (3H, s, OCH_3_); 5.53 (1H, s, CH); 6.68-7.22 (4H, m, CH_arom_).

### Antibacterial activity

The antibacterial activity of the synthesized compounds was determined by agar disk diffusion method against gram-positive (S. aureus bacteria) and gram-negative (E. coli bacteria) microorganisms. For this purpose, Briefly, Mueller Hinton agar culture medium with a thickness of 5 mm was prepared first. Then a suspension of the desired bacteria was prepared with 0.5 *McFarland* turbidity standards. Next, 50 µl of fresh bacterial culture was pipetted in the center of sterile petri dish containing the prepared culture medium and dispersed well by sterile swap. Next, filter paper disks containing a concentration of 2000 µg/ml of the synthesized products (3a-3j) are placed on the surface of the agar previously inoculated with the desired bacterium. The plates were then incubated for 24 hours at 37°C. The inhibition diameters zones formed around each disk were measured by using a ruler. Each experiment was repeated three times and the average diameter of the growth inhibition zone was calculated.

## Results and discussion

In order to optimize the reaction parameters, the catalytic activity of Zr(HSO_4_)_4_ in the synthesis of 1,8-dioxoctahydroxanthene derivatives under different reaction conditions was investigated. For this purpose, Reaction efficiency and reaction rate in different catalytic values and different solvents were investigated. Condensation reaction of benzaldehyde and dimedone was selected as a model reaction. As shown in Table 1, among the tested solvents such as water, THF, CHCl_3_, CH_3_CN_,_ CH_3_CH_2_OH and a solvent-free system, the best result was obtained after 40 min under solvent-free conditions in good yield (86%) (Table 1, Entry 7). When the same reaction was performed in the absence of the catalyst, the corresponding product was obtained in trace (<10%) (Table 1, Entry 9). However, the presence of Zr(HSO_4_)_4_ in the amount of more than 30 mol%, the efficiency and reaction speed did not improve (Table 1, Entry 8)

After determining the optimal reaction conditions, aldol condensation reaction between dimedone and benzaldehyde derivatives was performed in the presence of Zr(HSO_4_)_4_ catalyst and in optimal reaction conditions (Table 2). All the products were characterized by ^1^HNMR and IR spectra and compared with previous articles.

**Table 2:** Synthesis of 1,8-dioxooctahydroxanthene derivatives in the presence of Zr(HSO_4_)_4_ and in optimal reaction conditions.

Entry	R	Product	Time (min)	Yield %	Mp (˚C)	Mp (Lit)
**1**	C_6_H_5_	3a	40	86	201-203	205-206 (37)
**2**	*p*-CH_3_C_6_H_4_	3b	50	81	229-230	222-225 (38)
**3**	*p*- CH_3_OC_6_H_4_	3c	55	82	251-252	248-250 (37)
**4**	*m*- CH_3_OC_6_H_4_	3d	35	92	304-305	308-310 (38)
**5**	*p*-Cl C_6_H_4_	3e	40	88	240-241	237-238 (37)
**6**	*p*-NO_2_ C_6_H_4_	3f	35	94	230-032	226-228 (37)
**7**	*p*-OHC_6_H_4_	3g	60	80	254-255	250-251 (37)
**8**	2,4- di-Cl-C_6_H_4_	3h	30	95	239-241	247-248 (37)
**9**	*p*-(Me)_2_N C_6_H_4_	3i	60	72	295-297	-
**10**	*m*-Cl C_6_H_4_	3j	40	88	179-181	185-186 (39)

### Antimicrobial activities of 1,8-dioxo-octahydroxanthene derivatives

**Fig. 1:**
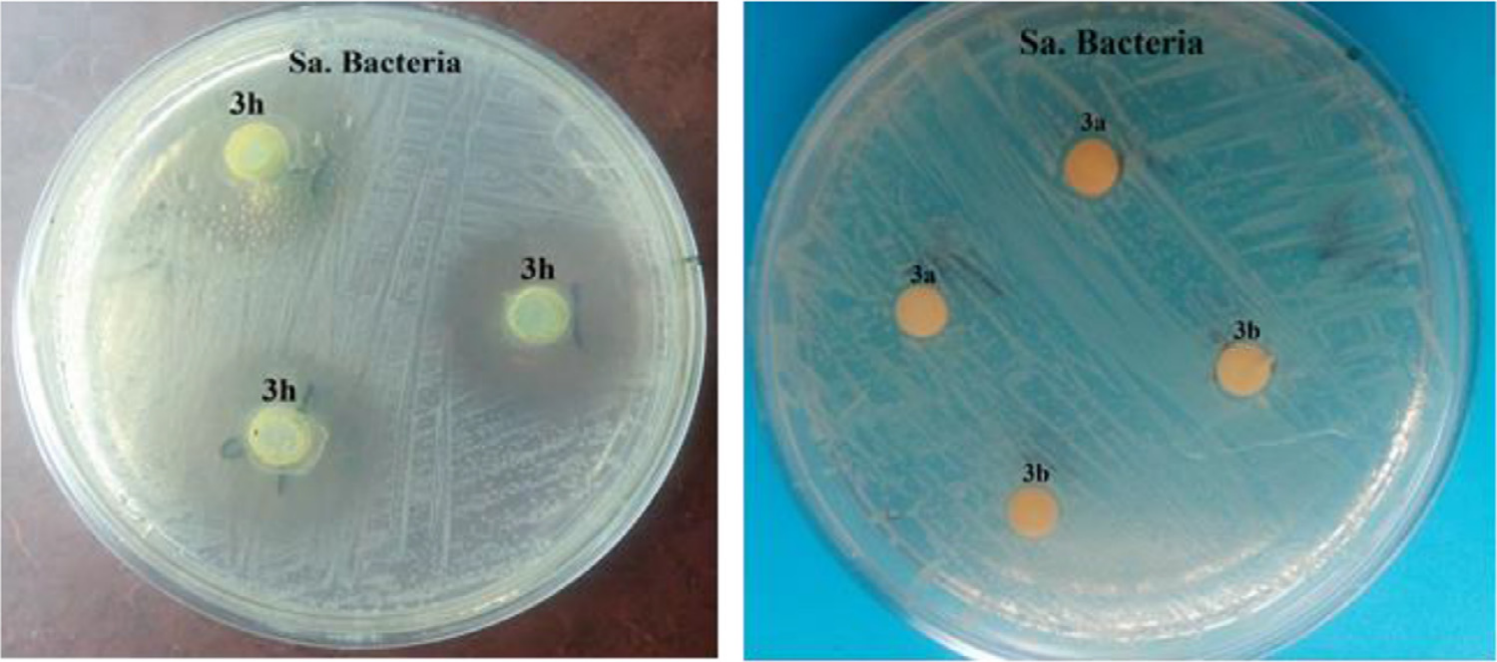
Perform antibacterial test by disk diffusion method against gram-positive (S. aureus bacteria) and gram-negative (E. coli bacteria) microorganisms.

The antibacterial properties of all synthesized compounds (3a-3j) were investigated by agar disk diffusion method against gram-positive (S. aureus bacteria) and gram-negative (E. coli bacteria) microorganisms. The results are reported in Table 3 and Fig. 1.

**Table 3:** Inhibition zones (mm) of synthesized 1,8-dioxooctahydroxanthenes derivatives against against gram-positive (S. aureus bacteria) and gram-negative (E. coli bacteria) microorganisms by the disc diffusion method.

Entry	Product	S. aureus	E. coli
1	3a	4*	0⁎⁎
2	3b	3.5	0
3	3c	0	0
4	3d	0	0
5	3e	0	0
6	3f	0	0
7	3g	0	0
8	3h	15	7.5
9	3i	4	0
10	3j	3	0

⁎ Numbers are reported in millimeters.

⁎⁎ The numbers reported are the inhibitions halos formation around the disk.

### Recovery of catalyst

**Fig. 2:**
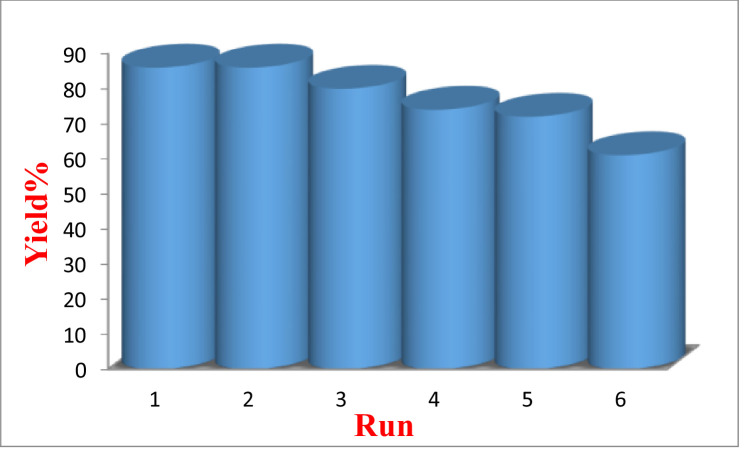
The reusability of Zr(HSO_4_)_4_ catalyst for the preparation of 3,3,6,6-tetramethyl-9-phenyl-1,8-dioxoocta hydroxanthene (3a).

Recycling of heterogeneous catalysts after the end of the reaction and its reusability with the least change in the reaction efficiency can be considered one of the most important advantages of this type of catalyst. In order to investigate the recovery of Zr(HSO_4_)_4_ catalyst, after each use of this catalyst, we washed it with ethanol and water and dried it in the oven and then participated in the same reaction. The results show that the catalyst can be used up to three run without significantly changing the reaction efficiency (Fig. 2).

**Scheme 1:**
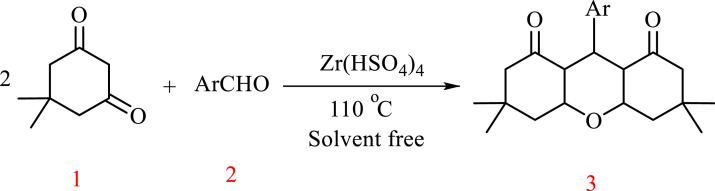
synthesis of 1,8-dioxooctahydroxanthenes.

**Scheme 2:**
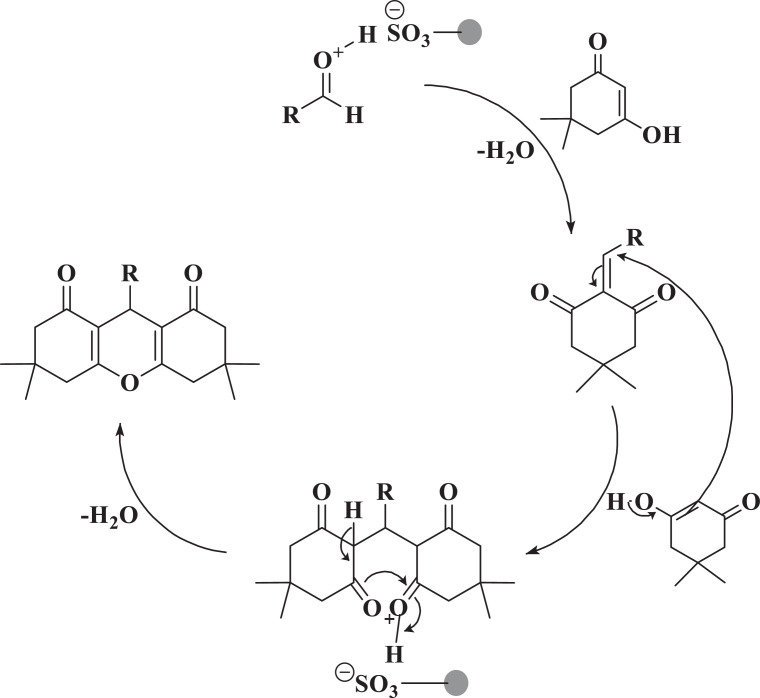
Proposed mechanism for the synthesis of 1,8-dioxooctahydroxanthene in the presence of Zr(HSO_4_)_4_ as catalyst.

The proposed mechanism is shown in Scheme 2. At first, SO_3_H groups, as an acidic agent, activates the carbonyl aldehyde group. Then dimedone attacks this intermediate. In the next step, by increasing Michael and the removal of a water molecule, the products are synthesized.

Table 4 shows a comparison between the methods reported in the articles and the method performed in this work.

**Table 4:** A comparison of the methods used in the articles and the method used in this work.

Entry	Catalyst	Conditions	Reaction time (min)	Yields%	Ref.
**1**	PEG	H_2_O/reflux	∼ 180	∼ 90	[39]
**2**	activated zinc metal and solid NH_4_C	Solvent free /MW	∼ 50	∼ 90	[40]
**3**	1-butyl-3-methylimidazoliumtetrafluoroborate (BMIF)	solvent free/∼200 ˚C	∼ 60	∼ 90	[41]
**4**	Fe nanoparticles loaded in zeolite X (Fe-X)	Solvent-free, 90°C	∼ 40	∼ 90	[42]
**5**	SO_3_H@Fe_3_O_4_ magnetic nanocatalyst	Solvent-free, 80 ˚C	∼ 20	∼ 90	[43]
**6**	Zr(HSO_4_)_4_	Solvent-free, 110°C	35-60	72-95	This work

## Conclusion

In summary, in this study we introduced compound Zr(HSO_4_)_4_ as a green, effective and recyclable catalyst for the one pot synthesis of various 1,8-dioxooctahydroxanthenes derivatives. Some advantages of this protocol include a simple reaction set-up, high products yields and elimination of toxic solvents.

## Declaration of Competing Interests

The authors declare that they have no known competing financial interests or personal relationships that could have appeared to influence the work reported in this paper.

## Data Availability

Data will be made available on request.

## Appendix Supplementary materials

Image, application 1
