# Supplementary material for: Zr(HSO4)4: Green, efficient and reusable catalyst for one-pot synthesis of 1,8-dioxooctahydroxanthene under solvent-free conditions
Source: MethodsX. 2022 Aug 27;9:101832. doi: 10.1016/j.mex.2022.101832 (PMC9450119; doi:10.1016/j.mex.2022.101832)
Supplement: Supplementary file 1 [file mmc1.docx]

**Supplementary material *and/or* Additional information:**

S1: Ft-IR spectra *3,3,6,6-tetramethyl-9-phenyl-1,8-dioxooctahydroxanthene* (3a)

S2: ^1^HNMR spectra *3,3,6,6-tetramethyl-9-phenyl-1,8-dioxooctahydroxanthene* (3a)

S2. Continued

S3: Ft-IR spectra *3,3,6,6-tetramethyl-9-(4-methylphenyl)-1,8-dioxooctahydroxanthene* (3b)

S4; ^1^HNMR spectroscopy *3,3,6,6-tetramethyl-9-(4-methylphenyl)-1,8 dioxooctahydroxanthene* (3b)

S4; Continued

S5; Ft-IR spectra *3,3,6,6-tetramethyl-9-(4-methoxyphenyl)-1,8-dioxooctahydroxanthene* (3c)

S6; S5; ^1^HNMR spectra *3,3,6,6-tetramethyl-9-(4-methoxyphenyl)-1,8-dioxooctahydroxanthene* (3c)

S6; Continued

S7; Ft-IR spectra *3,3,6,6-tetramethyl-9-(3-methoxyphenyl)-1,8-dioxooctahydroxanthene (3d)*

S8; ^1^HNMR spectra *3,3,6,6-tetramethyl-9-(3-methoxyphenyl)-1,8-dioxooctahydroxanthene* (3d)

S8; Continued
